# Longitudinal dynamics and clinical significance of acetylcholine receptor blocking antibodies in myasthenia gravis

**DOI:** 10.1007/s10072-026-09423-4

**Published:** 2026-09-25

**Authors:** Aigli G Vakrakou, Maria Belimezi, Eleni Strataki, Eleftherios Kontizas, Dimitra Tzavella, Dimitrios Panaretos, Emmanouil Angelakis, Vasiliki Zouvelou

**Affiliations:** 1 Neurology Department, Eginition Hospital, National and Kapodistrian University of Athens, ERN EURO-MND, 74 Vas. Sophias Ave, Athens, 11528 Greece; 2 Diagnostic Department, Hellenic Pasteur Institute, Athens, Greece; 3 Department of Statistics, School of Economic Sciences, University of Western Macedonia, Grevena, Greece

**Keywords:** Myasthenia gravis, Blocking antibodies, Binding antibodies, Generalized myasthenia gravis, Ocular myasthenia gravis

## Abstract

**Objectives:**

To investigate the longitudinal clinical relevance of acetylcholine receptor (AChR) blocking antibodies in myasthenia gravis (MG).

**Methods:**

We prospectively studied 47 AChR-positive MG patients with 121 serial antibody measurements over a mean follow-up of 2.8 years. AChR-binding antibodies were measured by a conventional radioimmunoprecipitation assay, whereas AChR-blocking activity was measured using a competitive radioimmunoassay. Disease severity was assessed using Myasthenia Gravis Foundation of America (MGFA) classification. Longitudinal associations were analyzed using generalized estimating equations (GEE).

**Results:**

Blocking antibodies (> 20% inhibition) were associated with MGFA severity across repeated observations in longitudinal GEE analysis (β = 0.045, *p* < 0.001), whereas binding antibodies were not significant. Among 13 patients with ocular-onset MG, all 8 who progressed to generalized disease were blocking antibody-positive, while none of the 5 patients who remained ocular tested positive. Blocking antibodies were detected in 90.5% of generalized MG patients, whereas all persistently ocular patients were negative.

**Conclusions:**

AChR blocking antibodies are associated with MG disease severity over time and may help identify ocular MG patients at risk of generalization, supporting their potential clinical relevance in MG.

**Supplementary Information:**

The online version contains supplementary material available at https://doi.org/10.1007/s10072-026-09423-4.

## Introduction

Myasthenia gravis (MG) is an autoimmune disorder of the neuromuscular junction characterized by antibodies targeting the acetylcholine receptor (AChR), leading to impaired neuromuscular transmission [1]. AChR antibodies are classified into binding, modulating, and blocking subtypes based on their pathogenic mechanisms [2–4]. While binding antibodies are well established in diagnosis and complement-mediated damage, the clinical significance of blocking antibodies remains controversial [5].

Blocking antibodies directly inhibit acetylcholine binding and are detected in approximately 50% of AChR-positive MG patients [6, 7]. Previous studies have yielded conflicting results regarding their clinical relevance. Some suggest association with generalized disease and thymoma [5], while others report inverse or weak correlations with disease severity [5, 8].

The present study aims to investigate the prevalence, longitudinal clinical associations across repeated measurements, and potential prognostic relevance of AChR-blocking antibodies in a cohort of MG patients from a single center.

## Methods

We conducted a prospective longitudinal study of 47 patients with acetylcholine receptor (AChR)-positive myasthenia gravis (MG) followed at a single tertiary neuromuscular center. Diagnosis was based on compatible clinical findings and positive AChR antibodies and/or electrophysiological testing. Patients positive for MuSK antibodies or seronegative for AChR antibodies were excluded. Clinical data included MG subtype, disease duration, thymoma status, treatment exposure, and Myasthenia Gravis Foundation of America (MGFA) classification.

Serum AChR-binding antibodies were measured using the conventional RiaRSR™ AChR-ab radioimmunoprecipitation assay (RSR Ltd., Cardiff, UK). AChR-blocking activity was measured using the competitive RiaRSR™ AChR blocking-ab Research Use Only assay supplied by RSR Ltd.; positivity was defined as > 20% inhibition. A total of 121 longitudinal antibody measurements were available for analysis. Detailed analytical procedures for both assays are provided in the Supplementary Methods.

Associations between antibody levels and MGFA severity were assessed using generalized estimating equation (GEE) models accounting for repeated within-patient measurements over time. Seven longitudinal GEE models were constructed, including combined and adjusted analyses incorporating age, sex, thymoma presence, and drug-naïve status. Statistical significance was defined as *p* < 0.05. Because MGFA is an ordinal clinical classification, cumulative link mixed models (CLMMs) with a patient-specific random intercept were additionally performed as a sensitivity analysis. Continuous variables are presented as mean (standard deviation [SD]), and categorical variables as number and percentage. Categorical variables were compared using Fisher’s exact test. Continuous variables were compared between groups using the Mann–Whitney U test.

Additional methodological details, longitudinal modeling procedures, treatment data, and supplementary analyses are provided in the Supplementary Methods.

## Results

Forty-seven patients with AChR-positive myasthenia gravis (MG) were included in the analysis, contributing 121 longitudinal antibody measurements of AChR binding and blocking antibodies over a mean follow-up of 2.8 years. Blocking antibodies (> 20% inhibition) were detected in 38/47 patients (80.9%). Generalized MG was present in 42/47 patients (89.4%), whereas 5 patients had persistently ocular disease. Blocking antibodies were detected in 90.5% of generalized MG patients, while all persistently ocular patients were negative.

Among 13 patients with ocular-onset MG, 8 progressed to generalized disease during follow-up; all were positive for blocking antibodies, whereas none of the 5 patients who remained ocular had detectable blocking antibodies. Blocking antibody levels were significantly higher in generalized versus ocular MG (*p* = 0.0004).

For all patients, antibody measurements were recorded along with corresponding MGFA severity scores. Each patient contributed between 1 and 8 measurements (mean = 2.53).

Longitudinal trends revealed distinct behaviors for the two antibody types (Supplementary Fig. 1). Binding antibodies remained relatively stable during the early disease years but showed increased variability later in follow-up. In contrast, blocking antibodies fluctuated during early disease before stabilizing over time. MGFA severity scores followed a similar pattern, with greater variability during the initial disease phase and relative stabilization thereafter (Table 1).

**Table 1:** Clinical and Demographic Characteristics of MG Patients: Blocking Antibodies Negative vs. Positive

Parameters tested	All patients	Blocking Abs Negative	Blocking Abs Positive	*p*-value (Blocking + vs. Blocking−)
Basic parameters				
Number of patients	47	9 (19.14%)	38 (80.85%)	N/A
AChR Binding Antibodies at first blood test (nmol/L) / mean (SD)	75.88 (181.4)	11.51 (20.19)	91.13 (199.0)	0.0008
AChR Blocking Antibodies at first blood test (% inhibition) / mean (SD)	38.42 (17.87)	10.52 (6.20)	45.03 (12.43)	< 0.0001
Sex (% female/% male)	51.1% female, 48.9% male	11.1% female, 88.9% male	60.5% female, 39.5% male	*p* = 0.010
Age at disease diagnosis (years) / mean (SD)	57.74 (19.07)	67.67 (11.27)	55.39 (19.87)	NS
Disease duration (years) / mean (SD)	8.681 (7.739)	7.296 (8.501)	9.009 (7.633)	NS
Disease classification				
Ocular (%)	5 (10.6)	5 (55.6%)	0	*p* < 0.0001
Generalised (%)	42 (89.4)	4 (44.4%)	38 (100%)	*p* < 0.0001
Generalized at onset (%)	34 (72.3)	4 (44.4%)	30 (78.9)	0.091
Secondary Generalization				
Transition from ocular-onset MG to Generalised disease (%)	8 (17)	0	8 for 8 (100)	*p* = 0.0008
Mean time of transition (years) / mean (SD)	6.871 (8.233)	N/A	6.871 (8.233)	N/A
Presence of Thymoma (%)	7 (14.89%)	0	7 (18.42%)	NS
Clinical severity scores at first blood test				
MGFA at first blood test	All *n* = 47	Blocking − *n*=9	Blocking + *n*=38	
0	9 (19.1%)	3 (33.3)	6 (15.8%)	NS
I	7 (14.9%)	4 (44.4)	3 (7.9%)	0.0184
IIa	4 (8.5%)	0	4 (10.5%)	NS
IIb	7 (14.9%)	1 (11.1%)	6 (15.8%)	NS
IIIa	3 (6.4%)	0	3 (7.9%)	NS
IIIb	6 (12.8%)	0	6 (15.8%)	NS
IVb	4 (8.5%)	1 (11.1)	3 (7.9%)	NS
V	3 (6.4%)	0	3 (7.9%)	NS
N/A	4 (8.5)	0	4 (10.5)	NS
Clinical severity scores at last follow up				
PIS				
PR	21 (44.7)	6 (66.7)	15 (39.5)	NS
MM	17 (36.2)	2 (22.2)	15 (39.5)	NS
Improved	4 (8.5)	0	4 (10.5)	NS
Unchanged	3 (6.4)	1 (11.1)	2 (5.3)	NS
Death	1 (2.1)	0	1 (2.6)	NS
Missing/ Unknown	1 (2.1)	0	1 (2.6)	NS
MG-ADL				
MG-ADL mean (SD)	0.595 (1.270)	0.1429 (0.378)	0.6857 (1.367)	NS

Abbreviations: *AChR* acetylcholine receptor, Abs antibodies, *SD* standard deviation, *NS* not significant, *MGFA* Myasthenia Gravis Foundation of America, *PIS* post-intervention status, *MG-ADL* Myasthenia Gravis Activities of Daily Living

To evaluate the association between antibody levels and disease severity over time, generalized estimating equation (GEE) models were applied with time since diagnosis modeled as a continuous variable. Binding antibodies were not significantly associated with MGFA severity (β = 0.003, *p* = 0.119) (Table 2, Model 1) and remained non-significant in combined analyses (Model 3). In contrast, blocking antibodies showed a significant longitudinal association with MGFA severity (β = 0.045, *p* < 0.001) (Model 2). This association remained significant after adjustment for time since diagnosis, age at diagnosis, and sex (β = 0.042, 95% CI 0.021–0.063, *p* < 0.001; Model 4), as well as in additional sensitivity analyses including thymoma presence and drug-naïve status (Models 5–7). In the fully adjusted model, blocking antibodies remained the only covariate significantly associated with MGFA severity (β = 0.043, *p* < 0.001).

**Table 2:** Longitudinal Regression Models Assessing the Association of AChR Antibodies with MGFA Severity

Model No.	Model Description	Significant Variables (*p*-value)	Meaning
1	MGFA_ordered ~ Binding_Ab + years_dx	β = 0.003, SE = 0.002, *p* = 0.119 (ns)	Binding antibodies alone with continuous time have limited predictive value
2	MGFA_ordered ~ Blocking_Ab + years_dx	β = 0.045, SE = 0.011, *p* < 0.001	Blocking antibodies significantly associated with MGFA severity
3	MGFA ~ Blocking_Ab + Binding_Ab + years_dx (GEE, combined)	Blocking: β = 0.043, SE = 0.011, *p* < 0.001 Binding: β = 0.003, SE = 0.002, *p* = 0.099 (ns)	Combined model: only blocking antibodies retain significance.
4	MGFA ~ Blocking_Ab + years_dx + Age + Sex (GEE, adjusted)	Blocking: β = 0.042, SE = 0.011, p < 0.001 Age: β = −0.005, p = 0.671 (ns) Sex: β = −0.524, p = 0.244 (ns)	Blocking Ab remain significant after adjustment for age and sex.
5	MGFA ~ Blocking_Ab + Binding_Ab + years_dx + Thymoma (GEE)	Blocking_Ab: β = 0.043, SE = 0.011, *p* < 0.001 Binding_Ab: β = 0.003, SE = 0.002, *p* = 0.099 (ns)	GEE confirms: blocking Ab independently significant; binding Ab non-significant (*p* = 0.099).
6	MGFA ~ Blocking_Ab + years_dx + age + sex + drug-naïve status, (GEE)	Blocking_Ab: β = 0.044, *p* < 0.001 drug-naïve status: *p* = 0.085	Blocking Ab remain significant after adjusting for age, sex, and treatment status
7	MGFA ~ Blocking_Ab + years_dx + age + sex + thymoma + drug-naïve status (GEE, fully adjusted)	Blocking_Ab: β = 0.043, *p* < 0.001 All other covariates: *p* > 0.08	In the fully adjusted model, only blocking Ab remain significant; all other variables are non-significant.

Abbreviations: *AChR* Acetylcholine Receptor, *GEE* Generalized Estimating Equations, *MGFA* Myasthenia Gravis Foundation of America (clinical severity classification, numeric 0–8), Gaussian family, exchangeable correlation. β: change in *MGFA* score (0–8) per unit predictor; *SE* standard error; ns: not significant, Ab: antibody, Binding_Ab: *AChR* Binding Antibodies (nmol/L), Blocking_Ab: *AChR* Blocking Antibodies (% inhibition), years_dx: Years since diagnosis (continuous time variable), p: p-value indicating statistical significance

**Fig. 1:**
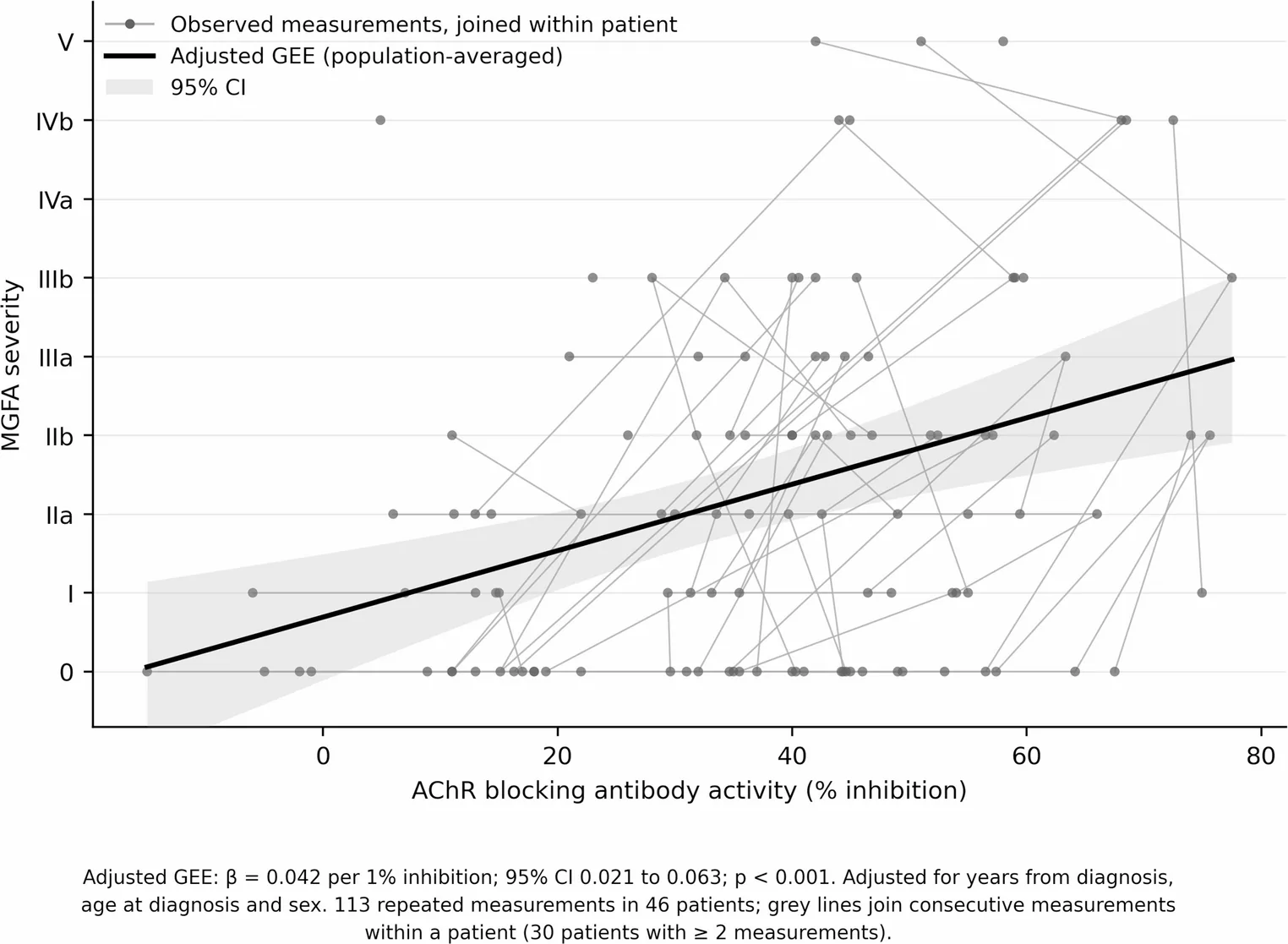
Adjusted population-averaged association of AChR-blocking antibody activity with MGFA severity. Observed repeated measurements of AChR-blocking antibody activity (% inhibition) and corresponding MGFA severity are shown, with grey lines connecting consecutive measurements within individual patients. The solid line represents the population-averaged relationship estimated using a generalized estimating equation (GEE) model adjusted for time since diagnosis, age at diagnosis, and sex; the shaded area represents the 95% confidence interval. The adjusted association was β = 0.042 per 1% increase in blocking activity (95% CI 0.021–0.063; *p* < 0.001). The model included 113 repeated measurements from 46 patients with all variable data

Figure 1 illustrates the adjusted population-averaged association between AChR-blocking antibody activity and MGFA severity. Observed repeated measurements are shown with consecutive measurements from the same patient connected by grey lines. The fitted GEE relationship was adjusted for time since diagnosis, age at diagnosis, and sex and remained significant (β = 0.042 per 1% increase in blocking activity, 95% CI 0.021–0.063, *p* < 0.001). The model included 113 repeated measurements from 46 patients with complete data for all variables included in the adjusted model.

Sensitivity analyses using cumulative link mixed models (CLMMs) yielded consistent results. Blocking-antibody activity remained significantly associated with greater MGFA severity (β = 0.0461, SE = 0.0142, *p* = 0.00125), whereas binding-antibody titers were not significantly associated with MGFA severity (β = 0.00218, *p* = 0.0843). In the model including both antibody measures, blocking-antibody activity remained significant (β = 0.0475, *p* = 0.00149), whereas binding-antibody titers remained non-significant (β = 0.00250, *p* = 0.0934) (data not shown).

## Discussion

In this study, we evaluated AChR-binding and AChR-blocking antibodies in a longitudinal MG cohort and identified distinct clinical roles. Binding antibodies were not significantly associated with MGFA severity in the longitudinal analyses. In contrast, blocking antibodies showed a consistent association with MGFA severity across repeated observations, including in adjusted analyses. Blocking antibodies were present in all patients who progressed from ocular-onset to generalized MG, whereas none of those who remained ocular were positive.

MG diagnosis relies on detection of AChR autoantibodies, most commonly using RIPA, which quantifies binding antibodies but does not directly detect blocking antibodies [9–11]. Previous studies suggest that both antibody types may contribute to disease expression, particularly when coexisting [7]. In our cohort, the mean blocking antibody inhibition rate in generalized MG (41.5%) was comparable to prior reports [5], while the overall prevalence (~ 80%) was higher.

Liu et al. reported that blocking AChR antibodies correlate with clinical severity, particularly in ocular MG, and may have greater predictive value than binding antibodies [5]. Our longitudinal findings extend this observation by demonstrating that this relationship persists over time and across disease stages. Notably, the prevalence of blocking antibodies in our cohort was higher than previously reported.

A key strength of this study is the availability of 121 antibody assessments obtained longitudinally across the cohort. The GEE models accounted for repeated observations from the same patient and showed a population-averaged association between higher blocking-antibody activity and greater MGFA severity. The concordant CLMM sensitivity analysis, which treated MGFA as an ordinal outcome and included a patient-specific random intercept, further supported the robustness of this association. Nevertheless, neither analysis establishes that changes in antibody levels within an individual patient necessarily parallel changes in MGFA severity over time. The cohort was highly selected, as all participants were AChR-binding antibody positive and most had generalized MG. Therefore, the high frequency of blocking-antibody positivity observed in this study should not be interpreted as an estimate of prevalence in an unselected MG population. In addition, competitive radioimmunoassays for AChR-blocking activity are technically demanding, and reported positivity rates may vary according to receptor antigen, assay design, positivity threshold, and cohort composition. Larger prospective studies are needed to validate the clinical relevance of blocking antibodies and their potential association with disease generalization.

## Conclusion

Although binding antibodies remain central to MG diagnosis, blocking antibodies show a more consistent association with disease severity. Their possible role in identifying patients at risk of generalization further underscores their clinical relevance.

## Supplementary Information

Below is the link to the electronic supplementary material.

Supplementary Material 1 (DOCX 37.0 KB)

Supplementary Material 2 (DOCX 20.8 KB)

Supplementary Material 3 (PDF 448 KB)

## Data Availability

Data will be made available on request.
