# Supplementary material for: Longitudinal dynamics and clinical significance of acetylcholine receptor blocking antibodies in myasthenia gravis
Source: Neurol Sci. 2026 Sep 25;47(10):809. doi: 10.1007/s10072-026-09423-4 (PMC13612679; doi:10.1007/s10072-026-09423-4)
Supplement: Supplementary file 1 [file 10072_2026_9423_MOESM3_ESM.pdf]

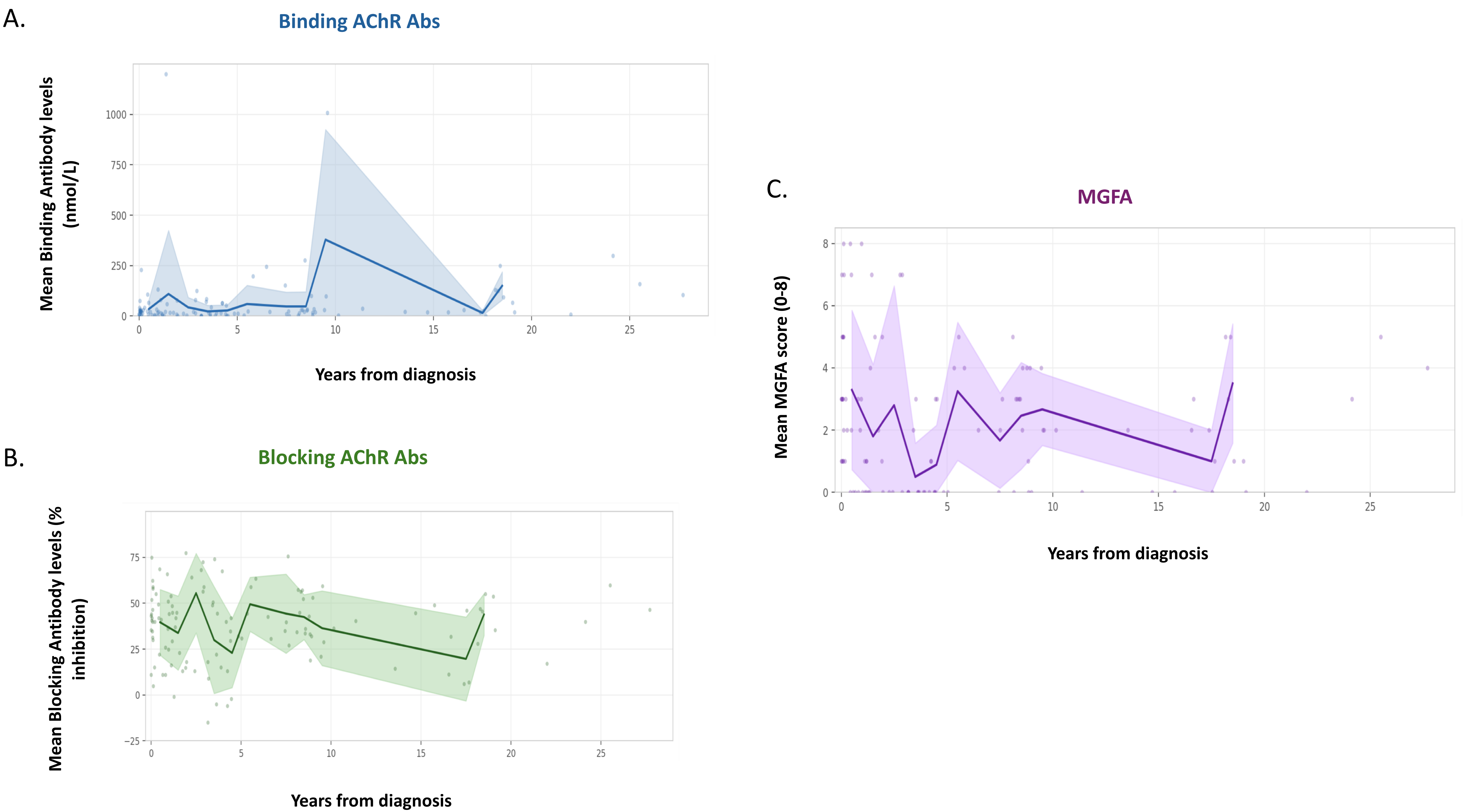

A.
Binding AChR Abs
Mean Binding Antibody levels (nmol/L)
0
250
500
750
1000
0
5
10
15
20
25
Years from diagnosis
B.
Blocking AChR Abs
Mean Blocking Antibody levels (% inhibition)
−25
0
25
50
75
0
5
10
15
20
25
Years from diagnosis
C.
MGFA
Mean MGFA score (0-8)
0
2
4
6
8
0
5
10
15
20
25
Years from diagnosis
