# Supplementary material for: Longitudinal dynamics and clinical significance of acetylcholine receptor blocking antibodies in myasthenia gravis
Source: Neurol Sci. 2026 Sep 25;47(10):809. doi: 10.1007/s10072-026-09423-4 (PMC13612679; doi:10.1007/s10072-026-09423-4)
Supplement: Supplementary file 2 [file 10072_2026_9423_MOESM1_ESM.docx]

**Supplementary Table S1. Comparison of AChR-blocking antibody studies in myasthenia gravis**

Studies are summarized according to MG population, blocking-antibody assay methodology and positivity threshold, and reported positivity or principal finding.

| **Study** | **MG population** | **Blocking-Ab method / cut-off** | **Positivity / main finding** |
| --- | --- | --- | --- |
| Present study | 47 AChR-positive MG; 42 generalized MG, 5 persistently ocular MG | RiaRSR™ AChR blocking-ab RUO assay; >20% inhibition | 38/47 (80.9%) overall; 38/42 (90.5%) generalized MG; all 5 persistently ocular patients negative |
| Besinger et al., 1983 (1) | 60 MG; prospective 2–5-year follow-up | Modified immunoprecipitation; human AChR + 125I-α-bungarotoxin; blocking titer in nmol/L | 29/60 (48%); correlated with changes in muscle weakness less often than binding-antibody titers |
| Howard et al., 1987 (2) | 349 acquired MG; blocking-antibody data available in 312 | Immunoprecipitation; solubilized human AChR + 125I-α-bungarotoxin; >25% abnormal | 161/312 (52%); 30% ocular, 52% mild generalized, 66% moderate–severe generalized MG |
| Kang et al., 2015  (3) | 35 MG; 10 ocular, 25 generalized | RIA; solubilized AChR + 125I-α-bungarotoxin + Sepharose-Con A; ≥15% | 24/35 (68.6%) |
| Aguirre et al., 2025 (4) | 75 MG with history of AChR-binding antibodies | Tecan AChR-blocking RRA; ≥45% | 37/75 (49.3%) |
| Liu et al., 2025 (5) | 148 MG; 76 ocular, 72 generalized | Commercial RiaRSR™ AChR blocking-ab kit (RSR Ltd., Cardiff, UK); >20% blocking defined as positive | 43.24% overall; 28.95% ocular; 58.33% generalized MG |
| Morcone et al., 2026 (6) | 30 AChR-positive generalized MG | Cell-based flow cytometry; clustered adult human AChR + AF488-α-bungarotoxin | 15/30 (50%) |

**Abbreviations:** AChR, acetylcholine receptor; MG, myasthenia gravis; RIA, radioimmunoassay; RRA, receptor radioassay; RUO, Research Use Only; α-BTX, α-bungarotoxin.

**References**

1.	Besinger UA, Toyka KV, Hömberg M, Heininger K, Hohlfeld R, Fateh-Moghadam A. Myasthenia gravis: long-term correlation of binding and bungarotoxin blocking antibodies against acetylcholine receptors with changes in disease severity. Neurology. 1983;33(10):1316-21.

2.	Howard FM, Jr., Lennon VA, Finley J, Matsumoto J, Elveback LR. Clinical correlations of antibodies that bind, block, or modulate human acetylcholine receptors in myasthenia gravis. Annals of the New York Academy of Sciences. 1987;505:526-38.

3.	Kang SY, Oh JH, Song SK, Lee JS, Choi JC, Kang JH. Both binding and blocking antibodies correlate with disease severity in myasthenia gravis. Neurological sciences : official journal of the Italian Neurological Society and of the Italian Society of Clinical Neurophysiology. 2015;36(7):1167-71.

4.	Aguirre F, Justo ME, Cialdella L, Paz ML. AChR-blocking antibodies and complement system dynamics: evaluating their interplay and clinical implications in myasthenia gravis. Neurological sciences : official journal of the Italian Neurological Society and of the Italian Society of Clinical Neurophysiology. 2025;46(4):1827-32.

5.	Liu K, Yang W, Liu X, Yu H, Zhu D, Li Y, et al. Association of blocking acetylcholine receptor antibodies with clinical subtypes and disease severity in myasthenia gravis. Clinica chimica acta; international journal of clinical chemistry. 2025;577:120449.

6.	Morcone V, Morroni J, Valenzano G, Marini S, Falso S, Marini M, et al. Functional Profiling of Acetylcholine Receptor Antibodies Informs Therapeutic Outcome of FcRn Inhibitor Therapy in Myasthenia Gravis. Neurology(R) neuroimmunology & neuroinflammation. 2026;13(4):e200616.
