# Supplementary material for: Longitudinal dynamics and clinical significance of acetylcholine receptor blocking antibodies in myasthenia gravis
Source: Neurol Sci. 2026 Sep 25;47(10):809. doi: 10.1007/s10072-026-09423-4 (PMC13612679; doi:10.1007/s10072-026-09423-4)
Supplement: Supplementary file 3 [file 10072_2026_9423_MOESM2_ESM.docx]

**Supplementary Methods**

**Patient Cohort**

Diagnosis of MG is based on fluctuating muscle weakness and the presence of one of the following two: (1) antibodies against nAChR or MuSK; or (2) abnormal repetitive nerve stimulation (RNS) or single-fiber electromyography (SF-EMG). Exclusion criteria for study enrollment were (i) patients positive for MuSK antibodies; (ii) patients seronegative for AChR [according to radioimmunoprecipitation assay (RIPA)]. Diagnosis was confirmed and clinical assessments of all patients were performed by the same physician (VZ).

The clinical factors analyzed include: age at diagnosis, years from diagnosis, type of disease (ocular versus generalized), generalization (whether disease transitioned from ocular to generalized form at least 3 months after symptom onset), thymectomy, thymoma presence, Myasthenia Gravis Foundation of America (MGFA) at serum testing, Post Intervention Status (PIS) at last follow-up and treatment modalities. Of the 47 patients in the cohort, 7 (14.9%) were diagnosed with thymoma; the histological types included WHO B1 (1 case), WHO B2 (3 cases), WHO B1/B2 (1 case), and WHO B3 (2 cases).

During the initial serum sampling for antibody testing, 11 out of 47 patients (23.4%) were drug naïve. During the follow up of all the studied cohort, drugs used for disease control were prednisolone in 45, Mycophenolate mofetil in 10, Azathioprine in 11, Rituximab in 14, Efgartigimod-alpha in 2, Ravulizumab in 2 and Rozanolixizumab in 2 out of 47 patients, with various combinations in some of them.

AChR Antibody Assays

AChR-binding antibodies were measured using the conventional RiaRSR™ AChR-ab radioimmunoprecipitation assay (RSR Ltd., Cardiff, UK). In this assay, fetal and adult human muscle AChR is pre-labelled with ^125I-α-bungarotoxin, followed by immunoprecipitation of antibody–AChR complexes with anti-human IgG. Results are expressed as AChR-binding antibody concentrations in nmol/L.

AChR-blocking activity was measured using the RiaRSR™ AChR blocking-ab assay, supplied by RSR Ltd. (Cardiff, UK) as a Research Use Only (RUO) assay and performed according to the manufacturer's instructions. Serum samples were incubated for 1 h at room temperature with a mixture of fetal and adult human muscle nAChR, followed by addition of ^125I-α-bungarotoxin and a further 2-h incubation. Antibodies interfering with α-bungarotoxin binding reduce the amount of radiolabelled toxin bound to AChR. The receptor-containing complexes were subsequently precipitated with Concanavalin A-Sepharose, centrifuged and washed, and radioactivity in the final pellet was measured using a gamma counter.

Blocking activity was calculated as:

Inhibitory activity (%) = [1 − ((cpm test sample − cpm NSB)/(cpm negative control − cpm NSB))] × 100

Values >20% inhibition were considered positive. Each reported blocking-antibody measurement was confirmed in more than two independent assay runs.

Thus, the two assays provide analytically distinct measurements. The competitive blocking assay quantifies percentage inhibition of ^125I-α-bungarotoxin binding to AChR relative to assay controls, whereas the conventional binding assay quantifies the concentration of AChR-binding antibodies in nmol/L.

**Statistical Analysis**

Continuous variables are presented as mean (standard deviation [SD]), and categorical variables as number and percentage. Group comparisons (e.g., sex, MG classification) were performed using non-parametric Mann–Whitney U tests for continuous or ordinal variables, and Fisher’s exact tests for categorical variables.

MGFA classification at each time point was recorded using the standard ordinal scale: 0 (no symptoms), I, IIa, IIb, IIIa, IIIb, IVa, IVb, V. This approach allowed for modeling the ordinal outcome (MGFA, scale 0-8, which combines both the class and subclass, allowing for a more nuanced assessment of disease severity) and intra-subject correlation simultaneously. To analyze longitudinal associations and account for repeated measures within patients Generalized Estimating Equations (GEE) models were used. Seven GEE models were constructed with time since diagnosis modeled as a continuous variable throughout: Model 1: Binding antibodies + continuous time. Model 2: Blocking antibodies + continuous time. Model 3: Both antibody types + continuous time (combined). Model 4 (adjusted): Blocking antibodies + continuous time + age at diagnosis + sex. Model 5: Both antibody types + continuous time + thymoma presence. Model 6: Blocking antibodies + continuous time + age + sex + drug-naïve status. Model 7 (fully adjusted): Blocking antibodies + continuous time + age + sex + thymoma + drug-naïve status. Statistical significance was determined at p < 0.05.

Because MGFA is an ordinal clinical classification, cumulative link mixed models (CLMMs) were additionally performed as a sensitivity analysis. MGFA was treated as an ordered categorical outcome and a patient-specific random intercept was included to account for repeated observations. Separate models evaluated blocking-antibody activity and binding-antibody titers with time since diagnosis modeled continuously, followed by a combined model including both antibody measures. The CLMM analysis was used to assess the robustness of the primary GEE findings when the ordinal nature of MGFA was explicitly modeled.

All statistical analyses were conducted using R (version 4.x), including GEE and CLMM modeling. Model-based graphical representations of the GEE analyses were generated in R. Graphical representations and additional descriptive statistics for the descriptive longitudinal analyses were generated using GraphPad Prism (version 8).
